# Micro- and Nanoplastics Breach the Blood–Brain Barrier (BBB): Biomolecular Corona’s Role Revealed

**DOI:** 10.3390/nano13081404

**Published:** 2023-04-19

**Authors:** Verena Kopatz, Kevin Wen, Tibor Kovács, Alison S. Keimowitz, Verena Pichler, Joachim Widder, A. Dick Vethaak, Oldamur Hollóczki, Lukas Kenner

**Affiliations:** 1 Division of Experimental and Laboratory Animal Pathology, Department of Pathology, Medical University of Vienna, 1090 Vienna, Austria; verena.kopatz@meduniwien.ac.at; 2 Department for Radiation Oncology, Medical University of Vienna, 1090 Vienna, Austria; 3 Center for Biomarker Research in Medicine (CBmed), microOne, 8010 Graz, Austria; 4 Comprehensive Cancer Center Vienna, Medical University of Vienna, 1090 Vienna, Austria; 5 Chemistry Department, Vassar College, 124 Raymond Avenue, Poughkeepsie, NY 12604, USA; 6 Department of Physical Chemistry, Faculty of Science and Technology, University of Debrecen, Egyetem tér 1, 4032 Debrecen, Hungary; 7 Department of Pharmaceutical Sciences, Division of Pharmaceutical Chemistry, University of Vienna, 1090 Vienna, Austria; 8 Institute for Risk Assessment Sciences, Department of Population Health Sciences, Faculty of Veterinary Medicine, Utrecht University, 3584 Utrecht, The Netherlands; 9 Department of Environment and Health, Vrije Universiteit Amsterdam, 1081 Amsterdam, The Netherlands; 10 Christian Doppler Laboratory for Applied Metabolomics, Medical University of Vienna, 1090 Vienna, Austria; 11 Unit of Laboratory Animal Pathology, University of Veterinary Medicine Vienna, 1210 Vienna, Austria

**Keywords:** polystyrene, micro-/nanoplastic, blood–brain barrier, biomolecular corona, computational uptake modeling

## Abstract

Humans are continuously exposed to polymeric materials such as in textiles, car tires and packaging. Unfortunately, their break down products pollute our environment, leading to widespread contamination with micro- and nanoplastics (MNPs). The blood–brain barrier (BBB) is an important biological barrier that protects the brain from harmful substances. In our study we performed short term uptake studies in mice with orally administered polystyrene micro-/nanoparticles (9.55 µm, 1.14 µm, 0.293 µm). We show that nanometer sized particles—but not bigger particles—reach the brain within only 2 h after gavage. To understand the transport mechanism, we performed coarse-grained molecular dynamics simulations on the interaction of DOPC bilayers with a polystyrene nanoparticle in the presence and absence of various coronae. We found that the composition of the biomolecular corona surrounding the plastic particles was critical for passage through the BBB. Cholesterol molecules enhanced the uptake of these contaminants into the membrane of the BBB, whereas the protein model inhibited it. These opposing effects could explain the passive transport of the particles into the brain.

## 1. Introduction

Micro-/nanoplastics (MNPs) are a growing concern, both for human health and the environment, due to their widespread distribution and potential harmfulness. Humans ingest a significant amount of MNPs through their diet [1], and plastic fragments are increasingly found in body fluids and tissues, such as blood and the placenta [2,3,4]. The definition of nanoplastic is still a topic of debate and varies from a range of 1000–1 nm to 100–1 nm [5,6,7]. To mitigate the potential harm of MNPs to human health and the environment, it is critical to limit exposure and reduce their use while continuing to study their effects [1,8]. MNPs can enter the body and cross impermeable barriers such as the intestinal mucosal barrier and the blood–brain barrier. The mechanism of transport of MNPs through these barriers is a complex process that depends on several factors such as particle size, charge, surface chemistry and the type of cell with which they interact [9]. For larger particles in the µm range, transport occurs through the binding of the particle to cell surface receptors [10] and the formation of a phagocytic shell that eventually fuses with lysosomes [11]. MNPs can also enter cells by endocytosis [12], in which the cell membrane engulfs the particle and brings it into the cell without the formation of a phagosome. On the other hand, particles less than 0.5 μm in diameter can potentially cross lipid bilayers through a process known as transcytosis [13], where the particle can diffuse through the lipid bilayer and exit at its other side without being engulfed into the cell. Nanoparticles applied for medicinal purposes revealed also passage via tight junctions for sizes of around 1.4 nm [14]. Further investigations are required to determine if this is also true for nanoplastic particles. However, the success of this process depends on the thermodynamics of the phase transfer from the aqueous medium into the membrane. The relative energy of the individual states must be similar for the transfer to occur at a reasonable rate [15]. If the particle is too stabilized or destabilized in the hydrophobic environment, the transport will be hindered by a barrier.

Earlier simulation studies [16,17,18,19,20,21] found that when nanometer-sized polystyrene (PS) particles absorb into the hydrophobic core of lipid bilayers, the polymer chains that make up the plastic particle may disentangle and form a network of macromolecules within the membrane. This process is dependent on the polymer type and the presence of cross-links and branches in the plastic material, but it can cause severe changes in the bilayer that can have physiological consequences. The calculations suggest that the hydrophilic polymer is significantly stabilized within the hydrophobic core of the membrane. Therefore, it would not be able to cross through this biological barrier effectively. The results of these experiments, however, raise the question: how can a particle with such thermodynamic properties cross the blood–brain barrier?

The surface of MNPs is crucial to their behavior in the human body, as it defines interactions between the particle and its environment. MNPs form a protein corona on their surface [22] which has been shown for nanoparticles of other materials to alter their interactions and toxicity. Walczyk et al., 2010 found that the protein corona, rather than the bare material properties of the particle, greatly influences interactions with the environment [23]. This is confirmed by recent studies with MNPs indicating that the protein corona acquired by plastic particles could significantly impact uptake and toxicity, such as in zebrafish [24] and mice [25]. Thus, the history of the coronated particle affects the outcome of experiments, emphasizing the need for rigorous methodologies when investigating the physiological effects of MNPs [24,25]. It is therefore essential to understand the changes that various coronae can introduce into the MNP–biomolecular interactions, especially regarding crossing the blood–brain barrier.

## 2. Materials and Methods

### 2.1. Materials 

Commercially available polystyrene micro- and nanoplastic particles were purchased from microparticles GmbH (Berlin, Germany). Particles were delivered in aqueous solution without modifier and used as received. In total, 3 different sizes were used (9.55 ± 0.13 µm particles, stained in blue; 1.14 ± 0.03 µm particles stained fluorescent red Ex/Em 530 nm/607 nm; 0.293 ± 0.008 µm particles stained fluorescent green, Ex/Em 502 nm/528 nm) and mixed at equal weight concentrations of 0.3 mg/size/dose in sterile water for application in mice.

### 2.2. Material Characterization and Stability 

Microparticles of 1.14 µm size were measured for their ζ-potential (mV), size distribution and polydispersity index (PDI) by means of a Zetasizer Pro (Malvern Pananalytical) and data were analyzed using ZS Xplorer software. The cuvettes were ZETASIZER Nano Series Disposable folded capillary cells (DTS1070) at 25 °C. The particles were delivered in an aqueous solution and were measured at a concentration of 0.5 mg/mL diluted in deionized water (0.55 µS), PBS and fully supplemented RPMI-1640 Media including 10% FBS and 1% L-glutamine. Deionized water, PBS and fully supplemented RPMI-1640 Media was filtered with a MILLEX-GV 0.22 µm filter to avoid particulate matter from the matrix. The 10 and 0.293 µm particles had to be excluded because the Zetasizer has a measurement range of 10–0.3 µm. For assessment of stability, MNPs of 1.14 and 0.293 nm size were incubated in simulated gastric fluid. Simulated gastric fluids were prepared as described within the U.S. Pharmacopoeia (0.16 M aq. HCl, 2 g/L NaCl and 3.2 g/L pepsin). Samples were incubated at 37 °C for 24 h and the supernatants were measured for fluorescence at Ex/Em 530 nm/607 nm and Ex/Em 502 nm/528 nm, respectively, in a Tecan Infinite M200 Plate Reader. 

### 2.3. In Vivo Experiments 

In total, 6 wild-type male C57Bl/6J mice were used in this proof of principle study (*n* = 2/group). Animals were bred in-house and kept under standard conditions (ambient temperature at 12/12 h light/dark cycle). Food and water were provided *ad libitum*. Experiments were conducted according to Austrian animal welfare legislation (license 2022-0.257.045), and experimental setups were approved by the local animal ethics committee. Briefly, mice were assigned randomly to 3 treatment groups and either left untreated (ctr) or gavaged with a single dose of 100 µL of MNP mixture as described above. Control and MNP-exposed mice were euthanized after 2 h or 4 h post gavage and mouse brains were harvested and processed according to a modified isopropanol protocol [26]. 

### 2.4. Fluorescent Microscopy Analysis 

Three micrometer tissue sections were cut and processed according to isopropanol protocol for immunofluorescence staining. Nuclei were counterstained with DAPI (Merck, Darmstadt, Germany). Images were taken at a Zeiss Axio Imager M2 microcope (63× objective). For image processing Zeiss Zen blue (version 3.5) was used.

### 2.5. Molecular Dynamics Simulations 

We chose 1,2-Dioleoyl-sn-glycero-3-phosphocholine (DOPC) as a model lipid; it is a predominant phospholipid in the human body, which has also been used extensively in simple and effective models for the blood–brain barrier [27]. The 2704 DOPC molecules in each membrane leaflet gave a 43.16 nm × 43.16 nm bilayer. The nanoplastic investigated here contained 4 chains of polystyrene, each with 100 styrene monomer units, folded together into a ca. 5 nm nanoparticle through a simulated annealing approach described elsewhere [28]. The effect of the particle size was not investigated here, as it has been demonstrated before that the absorption of the plastic nanoparticles into membranes was exothermic regardless of the size, i.e., qualitatively highly similar, although quantitatively the absorption energy changed [21]. It is reasonable to assume that this statement is valid unless the mechanism of the transfer through the bilayer changes to transcytosis, which happens at a much larger size threshold, at ca. 500 nm, beyond which point the absorption of the particle into the bilayer does not occur. Since in the present study only the direction of the absorption into the membrane will be discussed, as well as trends, and not the exact energetics, tracking the consequences of particle sizes is unnecessary.

The simulation boxes were generated by employing PACKMOL 20.10. [29,30]. Applying previously established coarse-grained models for the components of the system within the framework of the MARTINI force field [19,31], we used the GROMACS program package version 2020.3 for the simulations and the subsequent analysis [32,33,34,35,36]. The plastic particle was steered from the bulk of the liquid into the hydrophobic core of the membrane through umbrella sampling [37]. In this process, the membrane–plastic system was simulated in water, with the distance of the plastic to the lipid bilayer set to a defined value via an external harmonic potential. By repeating the simulations at different distances, the free energy profile of the phase transfer can be obtained using the weighted histogram analysis method (WHAM) [37,38]. The timestep in the simulations was set to 10 fs. Each system underwent a 60 ns equilibration in the NpT ensemble with a semi-isotropic Berendsen barostat set to 1 bar and a velocity rescale thermostat set to 310 K. The 40 ns production run was conducted at 310 K in an NVT ensemble. The same settings were used for the unbiased simulations, but the production run was conducted for 1 μs.

## 3. Results and Discussion

Polystyrene is a commonly used model plastic for studying the transfer of nanoplastic particles through membranes due to its widespread use and high environmental pollution levels, potentially leading to high exposure to the fragments. The size of the nanoparticles in the modeling part of this study was chosen to be ca. 5 nm, and a well-established coarse-grained model of PS was selected to make the calculations more efficient, along with a matching force field for the biomolecules in the system. For simulating the transfer of the plastics through the blood–brain barrier, DOPC bilayers were selected as a model membrane. In total, 4 models were used to study the role of the corona in the transfer: (1) pristine plastic, (2) a particle with a corona made of 100 cholesterol molecules, (3) a particle with a corona made of 150 cholesterol molecules and (4) a particle with a corona made of 40 protein molecules. Through these models, the importance of the corona in the transfer of the PS particle through the blood–brain barrier can be addressed. Protein coronae have been observed in experiments with various proteins, including human serum albumin [22,39]. Since in an earlier study we found that tryptophan has a large affinity to plastics in aqueous solutions [40], we chose a small protein featuring several of these amino acids to model the protein corona (PDB ID: 1LE1).

**Figure 1:**
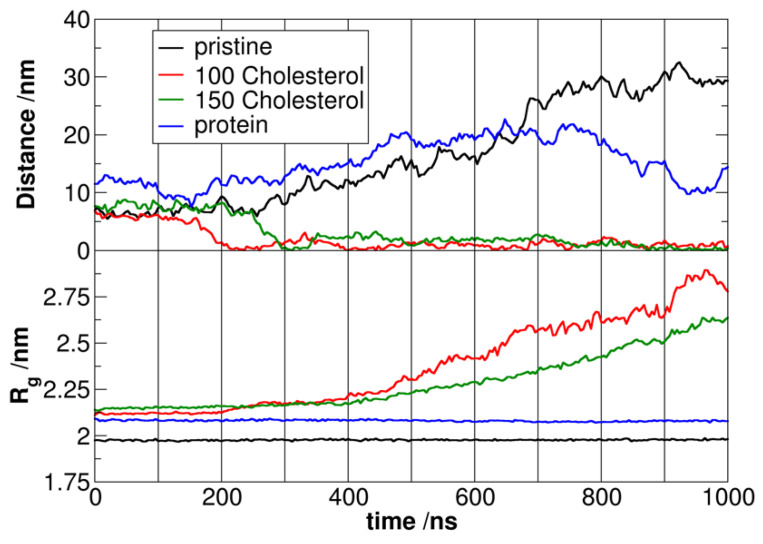
Development of the distances between the polystyrene particle and the DOPC bilayer in the four simulations (z component of the distance vector between the centers of mass, **above**), and that of the radius of gyration versus time (**below**).

The analysis suggests a strong interaction between the coronated plastic particle and its corona in the aqueous phase. This is evident from the swelling of the polymer chains in the presence of protein or cholesterol molecules, which is more extensive for the thicker corona of 150 cholesterol molecules (see the radius of gyration R_g_ values in Figure 1 in the first 200 ns). This swelling occurs because the non-polar molecules in the corona penetrate the plastic chains, leading to an increase in the surface and volume of the particle. The same effect can also be observed from the shrinking of the protein corona. Initial simulations showed striking differences, with pristine and protein-coronated plastic particles not entering the membrane within 1 μs. At the same time, those with cholesterol transferred spontaneously into the hydrophobic core of the membrane (Figure 1).

**Figure 2:**
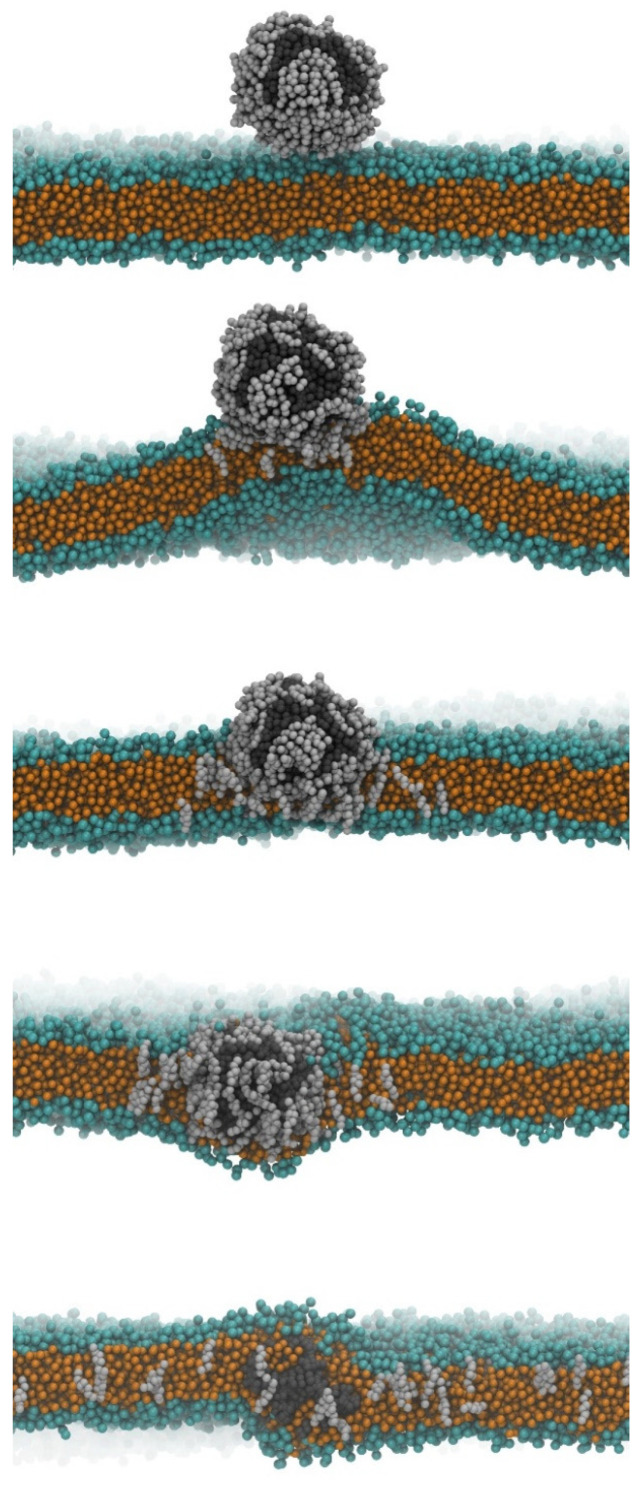
Snapshots of the simulations showing the entry of the plastic particle with a corona of 150 cholesterol molecules into the DOPC bilayer, a model blood–brain barrier (orange: hydrophobic moieties of the DOPC molecules; green: hydrophilic groups of the DOPC; dark grey: polystyrene; light grey: cholesterol). The nanoparticle approaches the membrane in the beginning of the simulation (**above**), and then spontaneously diffuses into its hydrophobic core, followed by the dissociation of its corona, and the disentanglement of the polymer chains by the end of the 1 μs run (**below**).

The mechanism for facilitating particle transfer was also identified in the two cholesterol simulations. The polar groups of cholesterol interacted with the DOPC in the membrane, resulting in a stable close contact as the coronated particle approached the bilayer (Figure 2). The interaction is amplified by the deformation of the lipid bilayer, forming a bulge towards the cholesterol corona molecules. As a result of this bulge, the closer lipid leaflet becomes looser, creating enough space for the cholesterol and plastic to diffuse into the membrane. The DOPC molecules rearrange to point their hydrophobic tails towards the incoming hydrophobic particles, leading to gradual encapsulation of the plastic by the hydrophobic tails. In contrast, the cholesterol molecules dissociate from the polymer and disperse in the lipid bilayer (Figure 2). Once the nanoplastic enters the hydrophobic core of the membrane, it is entirely surrounded and covered by lipids, leading to its slow dissolution and disentanglement of chains in the lipid bilayer, as previously observed. The behavior is dependent on the cross-links between polymer chains, as discussed in earlier studies. However, the membrane with the plastic in its hydrophobic core still retains some overall structure despite significant, potentially pathologically harmful differences compared to the neat bilayer. In the next set of simulations, the thermodynamics of the phase transfer process were calculated by steering the plastic particle into the membrane using umbrella sampling. For the pristine particle, an energy gain of −138 kJ/mol was found, in good qualitative agreement with previous data [39]. We found that the particle with the thinner cholesterol corona had a similarly exergonic phase transfer (−132 kJ/mol) to the pristine plastic, still with more cholesterol molecules, the driving force increased, exhibiting a free energy drop of −203 kJ/mol upon absorption into the membrane. In other words, the plastic without a corona or with a cholesterol corona can easily enter the blood–brain barrier but cannot exit it and thus cannot enter the neural tissue. In agreement, Notman and coworkers found that the entry of a pristine polystyrene nanoparticle into a DPPC membrane becomes more exothermic when cholesterol is present in the lipid bilayer [21]. The authors suggested that the reason for this finding may be that the cholesterol–polystyrene interplay is stronger than the DPPC–polystyrene interactions, although they also pointed out that the plastic does not seem to separate the cholesterol from the phospholipids. Our results here also suggest that there are more complicated effects at play, since cholesterol is in interaction with the polystyrene already before entering the membrane, thus—if it would come down only to the strength of the interactions—no increase in absorption energy should be observed.

Interestingly, for the plastic with a protein corona, the energy demand for entry was too high (+218 kJ/mol), indicating that it cannot enter the blood–brain barrier at all. The corona on the surface of the plastic apparently affects its thermodynamics for diffusion into the membrane. Therefore, it seems feasible that with the right combination of molecules, it can allow for the plastic to be absorbed into the membrane and cross over to the neural tissue. 

To underline these computational findings of MNP uptake into the brain, we performed short-term exposure experiments in mice with commercially available PS particles of 3 different sizes (9.55 µm, stained in blue; 1.14 µm particles stained fluorescent red Ex/Em 530 nm/607 nm; 0.293 µm particles stained fluorescent green, Ex/Em 502 nm/528 nm). The microplastic particles of the size 1.14 µm were characterized for their ζ-potential, average size and polydispersity index (PDI) in aqueous solution, PBS and fully supplemented cell culture media (Table 1). A similar trend of decreasing ζ-potential and increasing size and PDI were observed with increasing salt and component concentration within the matrix. The increased size is caused by the build-up of the protein corona in fully supplemented media. To test the stability of the MNPs, the particles were incubated in simulated gastric fluid to identify potential to leach out of the fluorophore at 37 °C for 24 h. No fluorescent signal was detected in the supernatant after the indicated time range. This result is in accordance with previously published leaching experiments [41].

**Figure 3:**
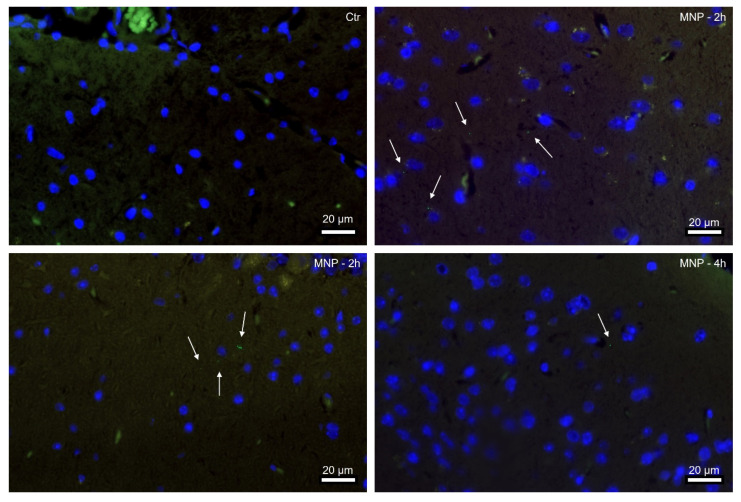
Nanometer-scale PS plastic particles were detected in mouse brain only 2 h after administration. Representative immunofluorescence images of male mouse brains, control (Ctr) and 2 or 4 h after single dose MNP gavage. Tissue sections were counterstained with Dapi (blue). Arrows indicate green fluorescent nanoplastic particles (0.293 ± 0.008 µm). Scale bar 20 µm.

For animal experiments, we administered MNP PS particle mixtures of 3 different sizes via oral gavage to wild-type mice and examined whether the particles can cross the BBB. To our surprise, we found specific nanometer-sized green fluorescent signals in the brain tissue of MNP-exposed mice after only 2 h, indicating that the 0.293 µm particles were already taken up shortly after administration (Figure 3). Numerous nanometer particles were detected at 2 h after gavage, whereas at 4 h the number was already lower. However, only the specific signal for the nanometer-scale PS particles was detectable. These findings together with the computer model underline that MNP PS particles can cross the gastrointestinal barrier and the BBB within a short time, but only 0.293 µm sized particles were able to be taken up from the gastrointestinal tract and to penetrate the BBB. This suggests that the size of the particles may be a critical factor in their ability to penetrate the BBB [42]. The BBB is an important barrier that protects the brain from toxins and other harmful substances, and its breach can lead to various neurological problems. Thus, short-term health effects of MNP will have to be considered, as MNP contamination in brain tissue can lead to cognitive impairment, neurological disorders and neurotoxicity [42], which may be attributed to the inhibition of acetylcholinesterase activity and altered neurotransmitter levels, both of which can contribute to behavioral changes [43]. Plastic particles at the nanoscale have a higher surface area to volume ratio, and they can be more reactive and potentially more harmful than larger microplastics [44]. Our in vivo findings are consistent with other experimental studies, e.g., in mice brain [45] and the brain tissue of zebrafish larvae [46]. 

Further research is needed to fully understand the health implications and toxicological mechanisms of MNP exposure and develop appropriate safety measures. Specifically, it will be important to investigate the long-term effects of MNP exposure, and the potential for accumulation and distribution of these particles in other tissues and organs. 

## 4. Conclusions

In recent years, plastic pollution has become an increasingly important environmental and human health issue. Although the environmental impacts of plastic pollution have been widely studied, the potential health consequences of plastic consumption on mammals including humans remain to be elucidated. The biomolecular corona is a layer of proteins and other biomolecules that can accumulate on the surface of plastic particles when they are exposed to biological fluids. Before entering the body, MNPs acquire an environmental or eco-corona consisting of biomolecules, organic matter and chemical and biological contaminants, contributing to the corona’s complex and variable composition [8]. The type of corona can therefore significantly impact their ability to enter the BBB and their overall toxicity.

Our computer models show that PS plastic particles are able to enter/cross the BBB depending on their specific surface corona, and in vivo mouse models verified these findings, showing accumulations of specifical signals of nanometer-sized PS particles in brain tissues as early as 2 h after exposure. The research also highlighted the importance of understanding the “corona” on the surface of plastic particles when assessing their toxicity. These new insights into the mechanisms for plastic particle transfer provide a valuable foundation for future research and policies aimed at mitigating their harmful effects on human health. Given the widespread use of plastics in our daily lives and the growing concern over the impact of microplastics on the environment and our health, there is an urgent need for more research in this field. By understanding the underlying mechanisms of plastic particle toxicity, we can develop policies and practices to reduce the risks associated with plastic consumption and protect human health.

## Figures and Tables

**Table 1:** Matrix-induced change of the zeta potential, average size and polydispersity index of 1 µm sized PS particles at pH 7.4 and 25 °C.

Particles		ζ-Potential (mV)	Average Size (nm)	PDI
1.14 ± 0.03 µm	H_2_O (0.55 µS)	−67.81	1206	0.01031
	PBS	−45.89	1259	0.07191
	RPMI-1640 (fs)	−14.03	1419	0.05526

fs = fully supplemented.

## Data Availability

The data presented in this study are available on request from the corresponding authors.
